# Mechanochemical Synthesis of Resveratrol–Piperazine Cocrystals

**DOI:** 10.3390/ma17133145

**Published:** 2024-06-27

**Authors:** Raul-Augustin Mitran, Simona Ioniţă, Daniel Lincu, Elena Mirabela Soare, Irina Atkinson, Adriana Rusu, Jeanina Pandele-Cuşu, Coca Iordache, Ingemar Pongratz, Mihaela Maria Pop, Victor Fruth

**Affiliations:** 1 “Ilie Murgulescu” Institute of Physical Chemistry Romanian Academy, 202 Splaiul Independentei, 060021 Bucharest, Romania; raul.mitran@gmail.com (R.-A.M.); simona.icf25@gmail.com (S.I.); daniel.lincu1113a@gmail.com (D.L.); ellena.mira19@gmail.com (E.M.S.); irinaatkinson@yahoo.com (I.A.); adyrusu2001@yahoo.com (A.R.); jeaninamirea@yahoo.com (J.P.-C.); 2 TeraCrystal SRL, Donat, No. 67-103, 400293 Cluj Napoca, Romania; coca.iordache@teracrystal.com; 3 Letavis AB, Västra Trädgårdsgatan 9, 111 53 Stockholm, Sweden; ingemar.pongratz@letavis.com

**Keywords:** resveratrol, piperazine, cocrystal, mechanochemical synthesis, up-scale

## Abstract

The 1:1 resveratrol–piperazine cocrystal was successfully synthesized and scaled-up to 300 g scale with the mechanochemical method, as a result of investigating key process parameters, namely the solvent and the grinding time. The use of water, ethanol or ethanol–water mixtures and reaction times up to 50 min were evaluated relative to the dry grinding process. Cocrystal formation and purity were monitored through X-ray diffraction and calorimetry measurements. The dry grinding resulted in an incomplete cocrystal formation, while the use of water or water–ethanol mixture yielded a monohydrate solid phase. Pure ethanol was found to be the optimal solvent for large-scale cocrystallization, as it delivered cocrystals with high crystallinity and purity after 10–30 min grinding time at the laboratory scale. Notably, a relatively fast reaction time (30–60 min) was sufficient for the completion of cocrystallization at larger scales, using a planetary ball mill and a plant reactor. Also, the obtained cocrystal increases the aqueous solubility of resveratrol by 6%–16% at pH = 6.8. Overall, this study highlights the potential of solvent-assisted mechanochemical synthesis as a promising new approach for the efficient production of pure resveratrol–piperazine cocrystals.

## 1. Introduction

The rise of antibiotic resistance is a global health threat in the 21st century. A part of the growth of antibiotic resistance is the overuse of antibiotics in both humans and farm animals [1]. It is estimated that 73% of worldwide antibiotics are used for livestock animals, such as pigs, cows and poultry, with pigs consuming the largest amount of antibiotics [2]. Plant extracts have recently gained traction as a potential functional alternative to antibiotics in livestock production [3]. The active ingredients are comprised of polysaccharides, polyphenols, alkaloids, etc. 

*Trans*-Resveratrol (3,5,4′-trihydroxy-*trans*-stilbene) is a natural polyphenol that exhibits a range of useful biological properties, such as antioxidant, anticancer, antiangiogenic, cardioprotective and immunomodulatory activities [4]. Resveratrol (Resv) is a promising bioactive compound to replace or reduce antibiotic use in meat production [5]. Despite its promising properties and success in research settings, resveratrol has found limited large-scale applications due to its limited aqueous solubility, which leads to poor bioavailability. The molecule also exhibits limited chemical stability due to isomerization or oxidation [6,7]. Solid-form modifications can be used to enhance the solubility of active pharmaceutical ingredients. These include amorphization [8], polymer dispersions [9,10,11], metastable polymorphs [12], salt formation [13] and cocrystallization [14]. Amorphous forms, solid dispersions and metastable polymorphs are not thermodynamically stable, while in some cases, such as for resveratrol, salt formation is not possible [15]. The unique physical properties exhibited by novel solid forms, such as cocrystals, can impact key pharmaceutical parameters, including storage stability, compressibility, density and dissolution rates and solubility, which are essential factors in achieving suitable bioavailability [16]. The development of new crystalline multicomponent forms of resveratrol has been documented in various studies aimed at improving its water solubility. Researchers have explored cocrystallization with compounds such as piperazine, 4,4′-bipyridine, phenazine, 1,10-phenanthroline, methenamine, DABCO, acridine, succinimide and N,N-dimethyl-4-aminopyridine [17,18]. In particular, resveratrol can form a thermodynamically stable cocrystal with piperazine (Pip), a small molecule with anthelmintic properties [17,18,19]. The 1:1 equimolecular resveratrol–piperazine cocrystal has been shown to have higher aqueous solubility than pristine resveratrol [18]. This cocrystal is thermodynamically stable and could be used to improve the therapeutic properties of the natural antioxidant. 

Cocrystals are typically obtained through the slow evaporation of a solution containing the precursor molecules or by precipitation [20]. While these methods are suitable for laboratory conditions, they are not optimal for the large-scale production of cocrystals. Solution evaporation and precipitation involve large amounts of solvent, long reaction durations and separation steps. Mechanochemical synthesis is an alternative route to solution-based cocrystal synthesis, offering higher energy efficiency, reduced solvent waste, high yields and an improved recovery of the final product [21,22]. These methods can involve limited amounts of solvent (solvent-assisted grinding) or no solvent at all (dry grinding, extrusion) [23]. Mechanochemical syntheses are often faster than solution-based methods, and they remove the necessity of processing steps, such as filtering. These methods can enhance yields and accelerate reaction rates compared to solution-based approaches [24].

Mechanochemistry unquestionably stands out as a valuable tool in cocrystal research, blending the quest for efficient and sustainable processing routes with the exploration of supramolecular synthons inaccessible via traditional solution-based methods [23].

The aim of this study was the mechanochemical synthesis of resveratrol–piperazine cocrystals, used as nutraceutical compounds. This study presents the first optimization study of the mechanochemical synthesis of resveratrol–piperazine cocrystals as well as the development of a batch cocrystallization process at a 300 g scale. Different synthesis conditions (the nature and amount of added solvent, reaction time) were investigated, and their influence on the cocrystal phase and purity were determined. The formation of the 1:1 (mol) cocrystal phase was monitored via differential scanning calorimetry (DSC) and powder X-ray diffraction (XRD). A solubility increase of up to 16% over that of resveratrol was determined through UV-Vis spectroscopy. Good thermal stability for the cocrystal was determined through thermogravimetry and in situ optical microscopy coupled with DSC. A reaction time of up to 30 min is sufficient for the completion of the reaction. Non-toxic solvents (water, ethanol) can be used to obtain a desired cocrystal phase with high purity.

## 2. Materials and Methods

### 2.1. Materials and Reagents

Resveratrol (Evolva SA, Reinach, Switzerland, purity > 98%), piperazine (Merck, Rahway, NJ, USA, purity ≥ 99%) and absolute ethanol (Merck, Rahway, NJ, USA, purity ≥ 99.9%) were used as purchased, without further purification. Ultrapure water (Millipore, Rahway, NJ, USA) was used in the syntheses. 

### 2.2. Conventional Synthesis of the Cocrystal

A 1:1 resveratrol: piperazine (mol) ethanolic solution was prepared by mixing 9 mL piperazine solution 2M with 90 mL resveratrol solution (0.2M). The solution was left to stand overnight at 25 °C. The yellow solids resulted after the complete ethanol evaporation were denoted “Conv”. 

### 2.3. Mechanochemical Synthesis

A 5 g mixture of resveratrol and piperazine 1:1 mol was prepared for each experiment. Solvent was then added for the solvent-assisted syntheses. The mixture was homogenized using a PM100 ball mill (Retsch, Haan, Germany) for a set amount of time (Table 1). The samples are denoted “*XX*-*YY*m/*Z*”, where *XX* denotes the solvent (N = no solvent, W = water, E = ethanol, EW = ethanol–water 1:1 *V*/*V*), *YY* represents the time in minutes, and *Z* is the solvent-to-solids ratio (mL g^−1^).

### 2.4. Mechanochemical Scale-Up

Scale-up experiments were performed using a 0.5 L planetary ball mill (Fritsch, Idar-Oberstein, Germany). Equimolar quantities of resveratrol (RESV) and piperazine (PIP) were weighted and added together with ethanol into the mill. The reaction mixture was stirred at 350 RPM and 20 °C for different periods, ranging from 0.5 to 4 h. The samples are denoted “SUx-yh”, where x is the experiment number, and *y* is the reaction time.

Another method of scaling-up was pursued using an IKA magic plant reactor (IKA, Wilmington, NC, USA), a laboratory scale process plant for batch production. Mixing was conducted by means of a special or propeller agitator, intensively mixing the product in radial and tangential directions. It can operate up to 150 °C and 2.5 bar, with a useful volume of 1–2 L. The rotation speed can be adjusted between 0–2000 rpm. The mixture (slurry consisting of 200 g resveratrol, 75.4 g piperazine and 620 mL ethyl alcohol) was homogenized for 6–8 min at 300 rpm, after which the speed was increased to 1700 rpm. Throughout the experiment, the temperature in the mantle was kept constant at 30 °C. In order to monitor the cocrystallization process, samples were taken for analysis every 10 min for 50 min. 

### 2.5. Characterization Methods

Powder X-ray diffraction (XRD) was carried out using a Rigaku Ultima IV diffractometer (Rigaku, Tokyo, Japan), with Cu-Kα radiation (λ = 1.5406 Å). Differential scanning calorimetry (DSC) analyses were performed using a Mettler Toledo DSC 3 calorimeter (Mettler Toledo, Columbus, OH, USA), under 80 mL min^−1^ nitrogen flow, at a heating rate of 10 °C min^−1^. Optical microscopy coupled with DSC was carried out using an Olympus SC50 Optical Microscope (Olympus, Tokyo, Japan), at a frame rate of 1 image per 1 °C. Thermogravimetric analyses (TGAs) were carried out using a Mettler Toledo TGA/SDTA851e thermogravimeter (Mettler Toledo, Columbus, OH, USA), under 80 mL min^−1^ air flow, at a heating rate of 10 °C min^−1^. Infrared spectroscopy (FT-IR) was carried out on a Thermo Nicolet 6700 spectrometer (Thermo Nicolet, Waltham, MA, USA), in KBr pellets. 

Solubility was determined using the shake-flask method. A sample excess (0.4 gL^−1^) was added to 5 mL water or phosphate buffer solution 0.2M, pH = 6.8 M, mechanically stirred for 24 h. The solids were filtered off, and the solution was analyzed via UV-Vis spectroscopy using an Agilent Cary 60 spectrometer (Agilent, Santa Clara, CA, USA). 

## 3. Results and Discussion

### 3.1. Influence of Solvent Addition

**Figure 1:**
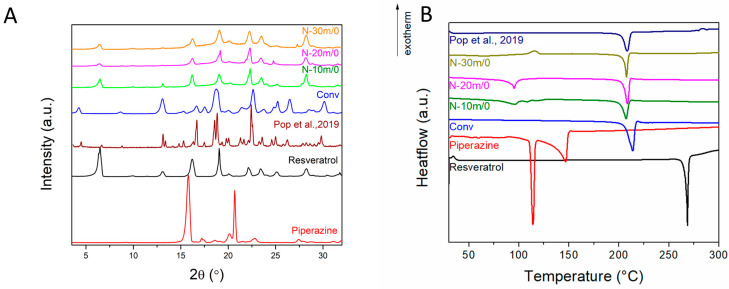
(**A**) XRD patterns and (**B**) DSC analyses of Resveratrol, piperazine, and cocrystals obtained through conventional (Conv) and dry grinding in comparison with the data from Pop et al., 2019 [17].

The resveratrol–piperazine cocrystal was prepared through the conventional, solution-based route in order to serve as a basis for comparison with the powders obtained from mechanochemical syntheses. The first mechanochemical syntheses were performed without solvent (i.e., dry grinding) for 10–30 min (Table 1). The X-ray diffraction (XRD) patterns of the conventional sample show the presence of diffraction peaks different from the starting precursors, indicating the complete formation of the desired cocrystal. The XRD patterns of the samples obtained through dry grinding indicate the presence of both the cocrystal and starting reagent phases (Figure 1A).

The melting points and heat of fusion values for each sample were determined through differential scanning calorimetry (DSC). The melting point of the conventional method cocrystal (205.6 °C) is different from that of resveratrol (267.8 °C) and piperazine (110.7 °C), indicating the complete formation of the cocrystal solid phase (Figure 1B). The endothermic event associated with the melting of the cocrystal can be noticed for the dry grinding samples (Figure 1B). Additional endothermic or exothermic peaks can be noticed for these samples between 75 and 150 °C, which could indicate that the reaction between the two precursor molecules occurs during the DSC measurements. The XRD and DSC data indicate the incomplete formation of the cocrystal phase through dry grinding.

**Figure 2:**
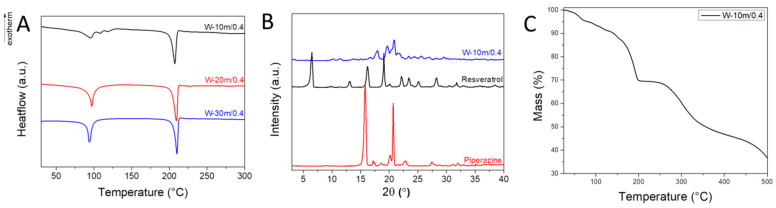
(**A**) DSC analyses of water-assisted grinding samples; (**B**) XRD and (**C**) TG analyses of W-10m/0.4.

Water-assisted grinding experiments were performed for up to 30 min (Table 1). The DSC measurements show the presence of a definite endothermic event between 80 and 110 °C, which could signify chemically bound water desorption (Figure 2A). Multiple endothermic events around 100 °C can be noticed for the sample subjected to 10 min grinding time, indicating incomplete cocrystal formation. The samples W-20m/0.4 and W-30m/0.4 show, however, only one water loss event. The XRD analyses of the water-assisted grinding experiments show a new diffraction pattern, different from the target cocrystal (Figure 2B). Thermogravimetric analyses (TGAs) of W-10m/0.4 exhibit a 4.8% wt. mass loss between 25 and 100 °C, which is in close agreement with the theoretical 5.4% wt. loss of an equimolar Resv:Pip:H_2_O = 1:1:1 composition of the sample (Figure 2C).

**Figure 3:**
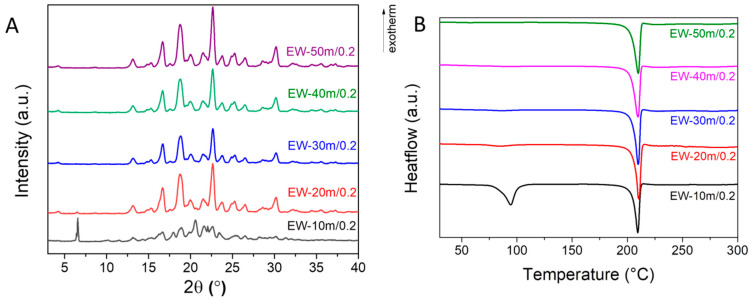
(**A**) XRD patterns and (**B**) DSC analyses of cocrystal samples obtained using ethanol–water-assisted grinding.

The ethanol–water (1:1 *V*:*V*) mixture was then investigated as a potential “green” solvent that could reduce the formation of the hydrate phase. The diffraction patterns show cocrystal formation after 20 min of reaction (Figure 3A). The DSC data show the formation of a hydrate after 10 min, as evidenced by the sharp endothermic event between 75 and 105 °C. The water is gradually lost when the grinding time is increased to 30 min (Figure 3B). The 1:1 (*V*/*V*) ethanol–water mixture can be used to obtain resveratrol–piperazine cocrystals through solvent-assisted grinding if the reaction takes enough time for complete water removal. 

**Figure 4:**
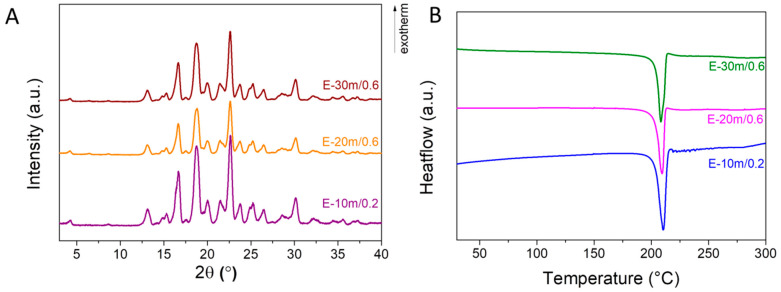
(**A**) XRD patterns and (**B**) DSC analyses of cocrystal samples obtained using ethanol assisted grinding.

Ethanol-assisted grinding experiments were performed for up to 30 min (Table 1). The diffraction patterns corresponding to the desired cocrystal can be noticed starting from 10 min (Figure 4A). Only the endothermic event associated with the melting of the cocrystal phase can be noticed in the DSC measurements (Figure 4B). All samples have similar heat of fusion values, varying from 158.0 to 163.8 Jg^−1^ (Table 1). This fact denotes that the cocrystal synthesis is complete after 10 min. It can thus be concluded that ethanol is the best solvent for solvent-assisted grinding. The results show that the solvent-assisted mechanochemical synthesis is a promising method for further up-scaling.

### 3.2. Thermodynamic Characterization of the Cocrystals

**Figure 5:**
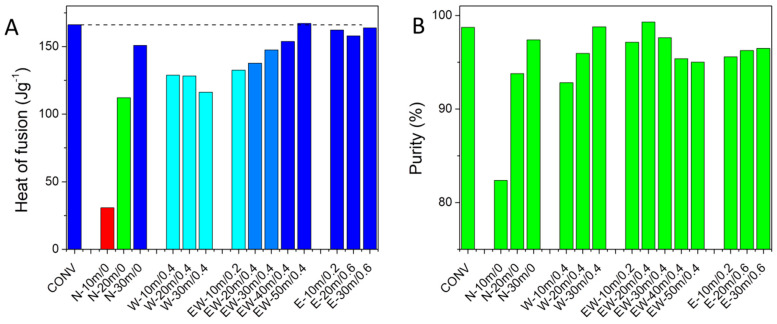
(**A**) Cocrystal heat of fusion values and (**B**) cocrystal purity computed from the melting point decrease using the cryoscopy equation. The dotted line in (**A**) represents the enthalpy of the convetional synthesis.

The heat of fusion values for the samples can be used to characterize the crystallinity and purity of the cocrystal phase with respect to unreacted coformers and residual solvent. A large variation in the heat of fusion values can be noticed, depending on the synthesis parameters (Table 1). The sample prepared using the conventional method has a melting enthalpy of 166.2 Jg^−1^, which is only exceeded by the EW-50m/0.4 sample (Figure 5A). Dry grinding yielded the lowest enthalpy values, followed by water-assisted grinding. These results indicate the incomplete formation of the desired cocrystal phase. The ethanol–water grinding experiments show an increase in enthalpy values with an increasing grinding time. Longer reaction times thus improve the yield and/or crystallinity of the samples. Ethanol-based samples exhibit high enthalpy values even at the lowest grinding time. 

The melting point (m.p.) of the cocrystals shows some variations. There are two likely mechanisms that can decrease the melting point of the cocrystal phase: particle size reduction in the 10–500 nm range can cause a change in m.p., as a consequence of reduced vapor pressure in capillary spaces [25], and the presence of impurities reduces the melting and the freezing points [26]. The cryoscopic equations were used to compute the purity of the cocrystal (Equations (1) and (2)) [27]. All samples except N-10m/0 have purities above 90% (Figure 5B). The highest purity value was obtained for the EW20m/0.4 sample, at 99.3%. The purity values for the water-based syntheses methods increase with reaction times, likely as a result of reducing the quantity of the hydrate phase.

(1) $$\Delta T=K_{f}\;m\;i$$

(2) $$K_{f}=\frac{R\;T_{f}^{2}\;M}{\Delta H}$$

Here $\Delta T\;\left({}^{\circ}C\right)$ is the m.p. depression of a sample with respect to the pure cocrystal; $K_{f}\;\left({K\;\mathrm{kg}\;\mathrm{mol}}^{-1}\right)$ is the cryoscopic constant; $m\;\left({\mathrm{mol}\;\mathrm{kg}}^{-1}\right)$ is the molality; i is the Van’t Hoff factor, equal to 2 for the Resv-Pip cocrystal; $R\;\left({J\;\mathrm{mol}}^{-1}K^{-1}\right)$ is the ideal gas constant; $M\left(g\;\mathrm{mol}{\;}^{-1}\right)$ represents the cocrystal molar mass; and $T_{f}\;\left({}^{\circ}C\right)\;$and $\Delta H\;\left({J\;g}^{-1}\right)$ are the freezing point and the heat of fusion values. 

Solvent-assisted grinding experiments performed using ethanol or ethanol–water solvents yielded the highest crystallinity, as evidenced by the heat of fusion values. While crystallinity increases with reaction time, the purity values have a maximum at a 20–30 min grinding time. Ethanol is the best solvent, since it also removes the possibility of obtaining secondary cocrystal phases, such as the hydrate phase obtained through water-assisted grinding. 

### 3.3. Physico-Chemical Characterization of the Resveratrol–Piperazine Cocrystal

**Figure 6:**
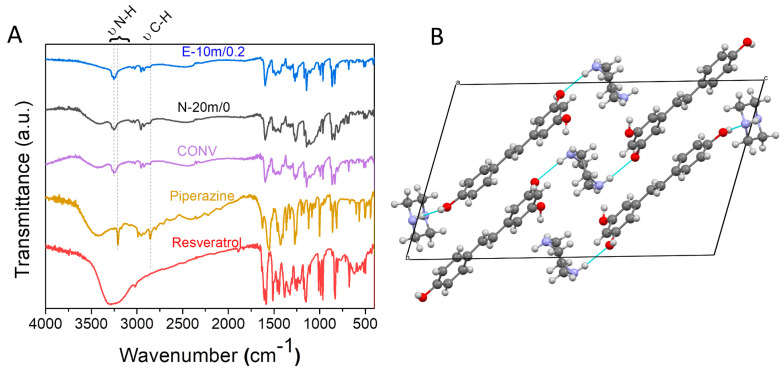
(**A**) FT-IR analyses of the starting conformers and representative cocrystals and (**B**) crystal structure depicting hydrogen bonding between the piperazine NH and resveratrol OH groups obtained from the structure with CCDC deposition number 1583810.

FT-IR spectroscopy was performed in order to assess the potential for unwanted chemical reactions during the mechanochemical synthesis of the cocrystal. The characteristic peaks of both resveratrol and piperazine in the 400–1700 cm^−1^ “fingerprint” region can be noticed for all cocrystals. The cocrystal peaks in the 400–1700 cm^−1^ range are the superposition of the resveratrol and piperazine spectra, with no significant shifts, indicating that no chemical changes had appeared after cocrystallization. The broad 3200–3600 cm^−1^ peak is associated with OH groups from resveratrol and adsorbed humidity. The 3208 cm^−1^ N-H stretching vibration of piperazine is shifted to 3252 cm^−1^ in the case of the cocrystals, which might be explained by the formation of intermolecular hydrogen bonds between the amine and resveratrol OH groups (Figure 6B). The FT-IR spectra thus show the preservation of the coformers and formation of the cocrystal phase. 

The solubility of selected cocrystals was compared with that of pure resveratrol. Solid samples were introduced in a limited volume of phosphate buffer solution (PBS), pH = 6.8. The slurry was filtered after 24 h and analyzed via UV-Vis spectroscopy. Resveratrol has a thermodynamic solubility of 63.0 ± 0.5 μg mL^−1^, in accordance with the literature data (Table 2) [28]. The cocrystals show increased solubility, between 69.9 ± 2.9 and 73.2 ± 0.9 μg mL^−1^. These differences might be explained by the different purities and crystallinity degrees of the samples. The cocrystal samples exhibit a 6%–16% increased solubility over the pristine resveratrol after 24 h. 

**Figure 7:**
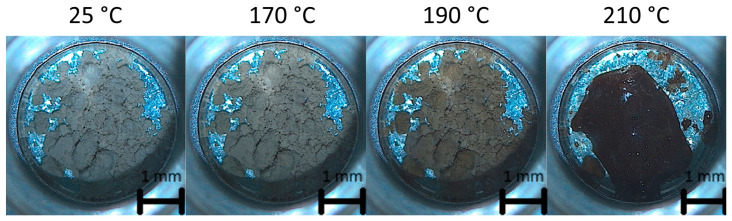
Optical microscopy images of the cocrystal sample prepared via ethanol-assisted grinding.

The thermal stability of the cocrystal sample was also investigated via optical microscopy coupled with DSC (Figure 7). The optical microscopy images show that the cocrystal is a light-yellow powder from 25 °C up to 170 °C. The cocrystal melts from 200 to 210 °C, where it transforms into a red-brown liquid. The analysis shows that the cocrystal undergoes thermal degradation after melting. The cocrystal sample changes color upon heating from 170 °C up to its melting point, from light yellow to red. The thermal stability of the resveratrol–piperazine cocrystal can be estimated to be at least 170 °C, based on the optical microscopy analysis. 

### 3.4. Scale-Up Experiments of the Resveratrol–Piperazine Cocrystal

**Figure 8:**
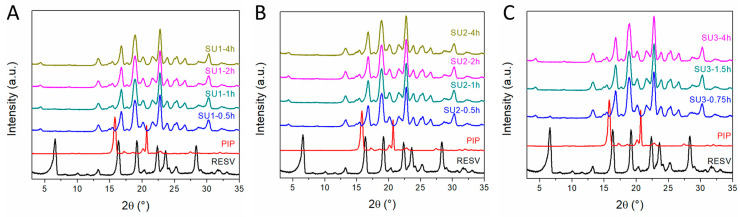
XRD patterns of (**A**) SU1, (**B**) SU2 and (**C**) SU3 scaled-up samples at different reaction durations.

The mechanochemical synthesis was initially scaled up using a planetary ball mill (Table 3). The reaction was carried out for up to 4 h, with samples periodically withdrawn and analyzed via XRD (Figure 8). The resveratrol–piperazine cocrystal is the main phase obtained for all samples and at all investigated reaction durations. Trace peaks of the starting materials can be noticed for the samples obtained after 0.5 or 0.75 h. A reaction time of 1 h is sufficient in all cases for the complete transformation of the starting materials into the desired cocrystal. The ethanol-assisted mechanochemical synthesis of the cocrystal could be successfully scaled up to ~100 g per batch.

The next scale-up method at the 300 g scale was performed in an IKA magic plant reactor. Table 4 presents the thermal analysis data for a series of cocrystal samples obtained in the presence of ethanol at different reaction times.

The thermal analysis results suggest that the samples of the resveratrol–piperazine slurry in ethanol exhibit consistent thermal behavior over the examined time intervals. The onset melting temperature and heat of fusion values show minimal variation, indicating stable thermal transitions. Overall, the slurry appears to have stable thermal properties under the studied conditions. The heat of fusion values for all slurry samples are similar to those obtained for neat grinding at 30 min, which might indicate lower crystallinity than the conventional synthesis.

**Figure 9:**
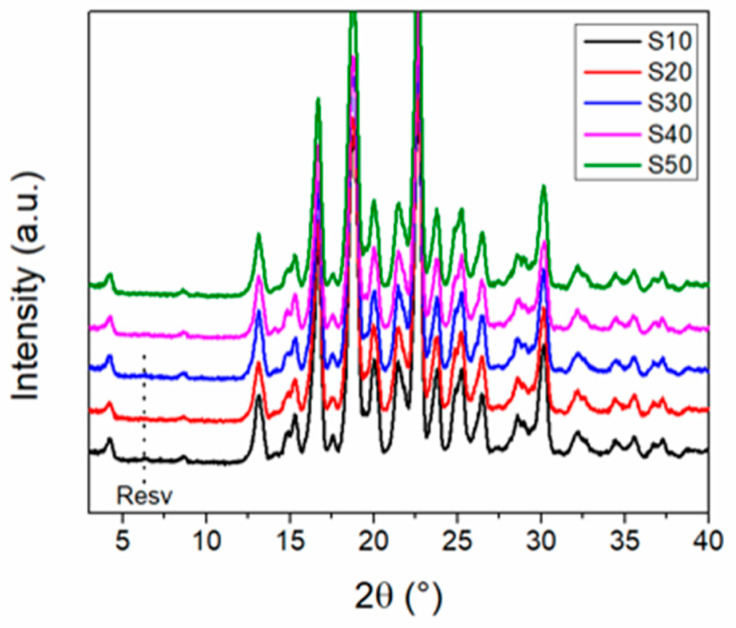
XRD patterns of the scaled-up samples (IKA magic plant reactor) at different reaction durations.

The resveratrol–piperazine cocrystal is the main phase obtained for all samples and at all investigated reaction durations (Figure 9). Trace peaks of the starting materials are observed in the samples obtained up to 30 min of reaction time. A reaction time of 40 min is sufficient for the complete transformation of the resveratrol and piperazine starting materials into the desired cocrystal. The ethanol-assisted mechanochemical synthesis method can be successfully scaled up to produce approximately 300 g per batch with this method.

## 4. Conclusions

This study reports the first optimization of the mechanochemical synthesis of the resveratrol–piperazine cocrystal, as well as the development of a batch cocrystallization process at the 300 g scale. In applying different synthesis conditions involving non-toxic solvents (water, ethanol and mixtures thereof) in combination with increasing grinding times, the optimal parameters for full conversion into the cocrystal were established at the laboratory scale and successfully transferred into a larger-scale cocrystallization process. Our results show that the use of pure ethanol is important for achieving the cocrystals without the formation of secondary crystalline phases, while a relatively fast reaction time (30–60 min) is sufficient for the completion of the cocrystallization at larger scale. The obtained resveratrol–piperazine cocrystal increased the aqueous solubility when compared with pristine resveratrol, thereby reinforcing the cocrystal’s use as a functional material. One can conclude that the solvent-assisted mechanochemical synthesis is a promising method for obtaining pure resveratrol–piperazine cocrystals with high yield. This method could prove to be a fast, economical and easy to scale-up method for the synthesis of the desired compound at industrially relevant scales.

## Figures and Tables

**Table 1:** Sample synthesis conditions and heat of fusion values for the melting of the cocrystal.

Sample	Solvent	Time (min)	V_solvent_/m_cocrystal_ (mL g^−1^)	ΔH (Jg^−1^)
Conv	-	-	-	166.2
N-10m/0	None	10	0	30.7
N-20m/0		20	0	112.2
N-30m/0		30	0	150.8
W-10m/0.4	H_2_O	10	0.4	128.8
W-20m/0.4		20	0.4	128.2
W-30m/0.4		30	0.4	116.3
EW-10m/0.2	EtOH:H_2_O 1:1 (*V*/*V*)	10	0.4	132.6
EW-20m/0.4		20	0.4	137.7
EW-30m/0.4		30	0.4	147.5
EW-40m/0.4		40	0.4	153.8
EW-50m/0.4		50	0.4	167.1
E-10m/0.2	EtOH	10	0.6	162.2
E-20m/0.6		20	0.6	158.0
E-30m/0.6		30	0.6	163.8

**Table 2:** Solubility data for resveratrol and representative cocrystal samples (data presented as average values ± standard deviation).

Sample	Solubility in PBS	
	(μg mL^−1^)	(%)
Resveratrol	63.0 ± 0.5	100 ± 1.1
N-10m/0	69.9 ± 2.9	111 ± 4.7
E-20m/0.6	73.2 ± 0.9	116.2 ± 1.7
CONV	67.0± 0.6	106.3 ± 1.3

**Table 3:** Scale-up conditions for the cocrystal synthesis.

**Sample**	**m_RESV_ (g)**	**m_PIP_ (g)**	**V_solvent_/m_cocrystal_** **(mL g^−1^)**
SU1	25.0	9.53	0.6
SU2	18.24	6.95	1.4
SU3	101.72	38.8	1.0

**Table 4:** Thermal analysis data for a series of samples obtained using IKA magic plant reactor at different reaction durations.

Sample	Time (min)	Onset (°C)	ΔH (Jg^−1^)
S10	10	204.9	146.6
S20	20	202.9	142.1
S30	30	204.4	144.0
S40	40	204.7	145.5
S50	50	203.4	148.0

## Data Availability

The data presented in this study are available on request from the corresponding author. The data are not publicly available due to non-disclosure of data.
